# Expression of geminin, p16, and Ki67 in cervical intraepithelial neoplasm and normal tissues

**DOI:** 10.1097/MD.0000000000007302

**Published:** 2017-06-30

**Authors:** Yan Xing, Chaojun Wang, Jie Wu

**Affiliations:** State Key Laboratory of Reproductive Medicine, Department of Obstetrics and Gynecology, The First Affiliated Hospital of Nanjing Medical University, Nanjing Medical University, Nanjing, China.

**Keywords:** cervical intraepithelial neoplasm, diagnosis, geminin, Ki67, p16

## Abstract

Geminin is a protein involved in cell cycle progression. We aimed to evaluate the diagnostic value of geminin expression in cervical intraepithelial neoplasm (CIN).

The expression of geminin, p16, and Ki67 was examined in 95 samples, including CIN1 (n = 45), CIN2/3 (n = 40), and normal cervical tissues (n = 10) by immunohistochemistry. The correlation between geminin or p16 expression and human papillomavirus (HPV) status was also evaluated.

Geminin expression was negative in all normal tissues and expressed in 13.3% of CIN1 and 90.0% of CIN2/3. P16 expression was demonstrated in 24.4% of CIN1 and 87.5% of CIN2/3. The corresponding Ki67 expression was 35.6% and 95.0%. The specificity of geminin for differentiating between CIN1 and CIN2/3 was 86.7%, while for p16 and Ki67 the corresponding values were 75.6% and 64.4%. The sensitivity of geminin, p16, and Ki67 was 90.0%, 87.5%, and 95.0%, respectively. The positive predictive value (PPV) and accuracy of geminin were higher than p16 and Ki67. In addition, geminin expression showed a weak correlation with HPV status, but there was no association between p16 expression and HPV status.

These results suggested that geminin had a high degree of sensitivity and specificity in determining CIN2/3. In addition to p16 and Ki67, geminin might be used as a new biomarker to distinguish between CIN1 and CIN2/3.

## 1 Introduction

Invasive cervical carcinoma is preceded by preinvasive cervical intraepithelial neoplasm (CIN). The most recent classification system of World Health Organization (WHO) applied low-grade squamous intraepithelial lesion (LSIL) and high-grade squamous intraepithelial lesion (HSIL). However, CIN is previously classified into CIN1, CIN2, and CIN3 on the basis of the extent of epithelial involvement.^[1]^ CIN1 is usually not precancerous and does not require treatment. The progression rate of CIN2/3 to invasive cervical cancer is 10% to 40%.^[2,3]^ Given the malignant potential of CIN2/3, it is important to have an accurate grading of CIN and to treat these patients properly. High-risk human papillomavirus (HPV) types, including HPV-16 and HPV-18, have been identified as the major cause of cervical carcinomas and precancerous lesions. Currently, the incidence rate of cervical cancer has decreased through screening by cytological Pap smear testing and HPV testing.^[4]^ HPV-18, one common reason for CIN2/3, is poorly detected by cytology and colposcopy.^[5]^ Furthermore, HPV testing is more sensitive than cytology for predicting CIN2/3, but is of less specificity.^[6]^ Histological diagnosis of cervical biopsies is regarded as “gold standard.” However, the interobserver and intraobserver variabilities are high.^[1]^ For these reasons, it is important to have an accurate diagnosis and prediction of progression risk for clinical management of patients with CIN.

P16 (also known as p16INK4a), a cyclin-dependent kinase inhibitor, is a cell-cycle regulatory protein. The high-risk HPV E7 oncoproteins bind and inactivate pRb, leading to abnormal cell proliferation. P16 is an accurate marker for this event. However, p16 is a less specific marker because CIN1 and CIN2/3 sometimes show similar p16 expression, and p16 also can be found in inflammatory cervical lesions.^[7,8]^ Ki67 is a marker of cell proliferation. A number of studies have shown that an increased expression of Ki67 is correlated with higher cervical CIN grade and is a highly sensitive biomarker for differentiating between CIN1 and CIN2/3, but Ki67 immunostaining is variable and less specific in many cases.^[9,10]^ Currently, p16 and Ki67 have been proposed to identify persistent infections with high-risk HPV types of cervical precursor lesions. Despite the value of these 2 complementary alternative biomarkers, staining of p16 and Ki67 is not sufficient to give a definite diagnosis and an accurate differentiation in some cases.

Geminin is located on the sixth chromosome, and the protein has about 209 amino acids. Geminin is an important component of the licensing system that is involved in cell cycle progression.^[11]^ Alterations in geminin expression are associated with cell proliferation, differentiation, and development. The expression of geminin is increased as the cell cycle progresses and downregulated when the cells exit the cell cycle.^[12]^ A great number of studies have reported that geminin plays a vital role in cancer pathophysiology and development. Differential geminin expression is associated with various cancers, including breast cancer, colorectal carcinomas, and small lung adenocarcinoma.^[13–15]^ Suppression of geminin can inhibit cancer cell proliferation without affecting the normal cells.^[16]^ Using high-density microarrays, Martin et al^[17]^ have identified several genes involved in cell cycle regulation that are differentially expressed in premalignant and malignant cervical disease, including geminin, p16, minichromosome maintenance complex component (MCM) 3, and MCM5. Real-time polymerase chain reaction (PCR) and immunohistochemisitry were used to confirm microarray results. This study suggested that geminin might play a role in prediction of progression of cervical precursor lesions.

In this study, we aimed to evaluate and compare staining pattern for geminin, p16, and Ki67 expression in normal cervical tissues, CIN1 and CIN2/3 to determine whether geminin can serve as an additional marker for the diagnosis of CIN2/3. In addition, we investigated whether geminin could discriminate between HPV-positive and HPV-negative precursor lesions.

## 2 Methods

### 2.1 Patients and controls

A total of 95 cervical samples were recruited from the First Affiliated Hospital of Nanjing Medical University between January 2015 and January 2016, including 10 normal cervical tissues, 45 CIN1, and 40 CIN2/3. The diagnosis of CIN was peer-reviewed according to the International Federation of Gynecology and Obstetrics criteria. All hematoxylin-eosin stained slides were reviewed by 2 independent pathologists. Exclusion criteria are patients who had a clinical record of radiotherapy or chemotherapy. The clinical characteristics of the subjects are summarized in Table 1. This research project was approved by the Ethics Committee of the First Affiliated Hospital of Nanjing Medical University. Informed written consent was obtained from all subjects.

**Table 1:**
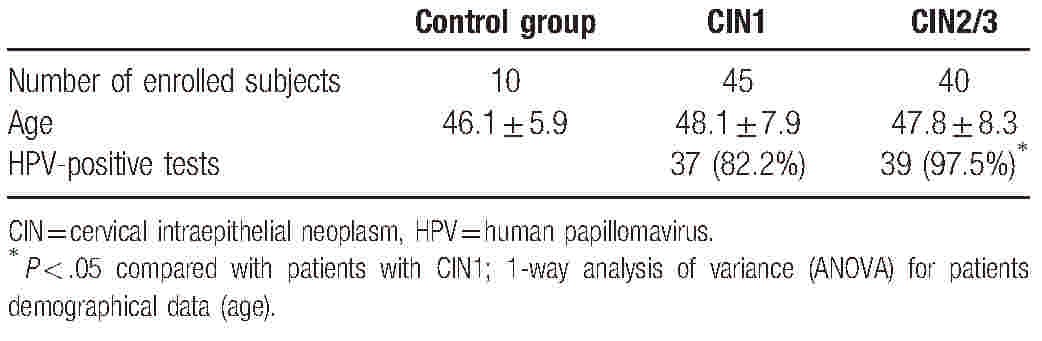
Patient clinicopathologic characteristics.

### 2.2 HPV detection

HPV DNA was detected by the Hybrid Capture II System. The scrapes were tested for the presence of high-risk HPV types 16, 18, 31, 33, 35, 39, 45, 51, 52, 56, 58, 59, and 68.

### 2.3 Immunohistochemistry analysis

All formalin-fixed paraffin-embedded sections were cut at 5 μm thickness and then dewaxed through xylene and dehydration with graded ethanols. The tissue sections were treated with 3% hydrogen peroxidase solution for 20 minutes to block endogenous peroxidase activity. Antigen retrieval was performed in 10 mmol/L of citrate buffer (Dako, Carpinteria, CA). The sections were then incubated overnight with a monoclonal mouse antibody to geminin (Abcam, Cambridge, MA) at 1:100 dilution, a monoclonal rabbit antibody to p16 (Abcam, Cambridge, MA) at 1:100 dilution, and a monoclonal rabbit antibody to Ki67 (Abcam, Cambridge, MA) at 1:500 dilution, respectively. Subsequently, tissue sections were counterstained with hematoxylin and then examined by light microscopy. For negative controls, substitution of primary antibody with TBS was run simultaneously.

### 2.4 Evaluation of geminin, p16, and Ki67 expression

Two independent authors (YX and WC) scored the expression of immunostaining slides. First, 10 normal cervical tissues were evaluated for geminin expression because there is not yet a validated cutoff value for positivity of geminin staining. We investigated that geminin expression was absent in most normal tissues. Therefore, immunostaining with Ki67 and geminin was considered positive when more than 5% of the cells showed strong positive nuclear staining. The immunoreactivity of p16 was judged as positive when there was a diffuse staining in both nuclear and cytoplasm of basal or parabasal cells.^[18]^ The cutoff value for interpretation of p16 staining is 5%. All the markers were classified into 4 groups: 0 (all cells negative), 1+ (positive staining in 6–25% of cells), 2+ (positive staining in 26–50% of cells), or 3+ (positive staining in more than 50% of cells). For the statistical analysis and evaluation of sensitivity, specificity, positive predictive value (PPV), negative predictive value (NPV), and accuracy, we subdivided the staining results into 2 groups: nuclear or cytoplasmatic immunoreactivity in less than 25% (all samples that were scored 0 and 1+) or more than 25% (all samples that were scored 2+ and 3+).

### 2.5 Statistical analysis

Statistical analysis was performed using SPSS software package, version 21.0 (IBM Corp, Armonk, NY). Data in this study are expressed as means ± standard deviation (SD). Chi-squared tests were used to differentiate between CIN1 and CIN2/3 and 1-way analysis of variance (ANOVA) was used to analyze the age among groups. Spearman rank correlation coefficients were analyzed to investigate the possible correlations between HPV status and geminin/p16 expression. Sensitivity, specificity, PPV, NPV, and accuracy were calculated on the basis of geminin, p16, and Ki67 staining results by the formulas of Galen and Gambino: Sensitivity=True Positive/(True Positive+False Negative); Specificity=True Negative/(False Positive+True Negative); PPV=True Positive/(True Positive+False Positive); NPV=True Negative/(False Negative+True Negative); Accuracy=True Positive+True Negative/(True Positive+False Positive+False Negative+True Negative). All statistical tests were 2-tailed and a *P* value of less than .05 was considered to be significant.

## 3 Results

Table 1 gives an overview of the clinicopathological characteristics of all 95 cases. All slides were re-reviewed and the histopathologic results were consistent with the initial diagnosis. There were no differences in patient age among the groups (*P* > .05). The HPV in patients with CIN1 was more frequently detected compared with the patients with CIN2/3 (*P* < .05).

The immunohistochemical staining results for geminin, p16, and Ki67 are summarized in Table 2. Geminin expression was absent in all of the normal cervical samples. In 1 (10%) normal cervical sample, geminin was immnoreactive in the cytoplasm of some cells, but it was not present in more than 5% of the cells. Geminin staining was present in less than 25% of the cells in 39 (86.7%) of 45 CIN1 and scored as 2+ in the other 6 (13.3%) CIN1. Our study demonstrated that the expression of p16 and Ki67 was completely negative in normal tissues. Staining with p16 was positive in more than 25% of the cells in 11 (24.4%) of the CIN1 and 35 (87.5%) of the CIN2/3, while Ki67 expression was found in 16 (35.6%) CIN1 and 38 (95.0%) CIN2/3, respectively. The expression of geminin, p16, and Ki67 was all significantly different between CIN1 and CIN2/3 (*P* < .05).

**Table 2:**
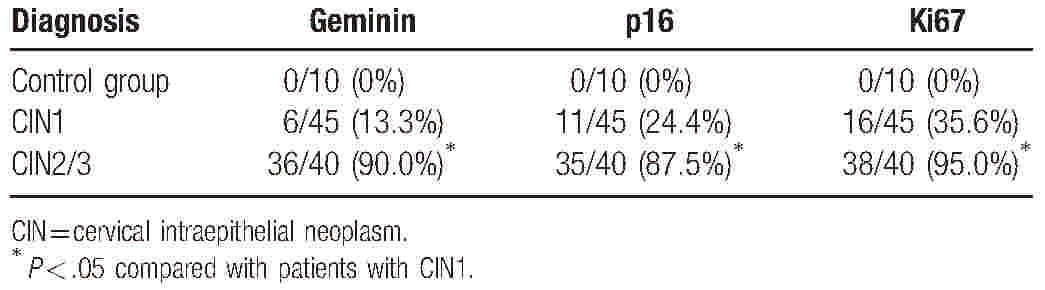
Results for geminin, p16, and Ki67 expression according to histopathology; the number of samples that were scored as 2+ or 3+.

Then, we studied the performance of geminin, p16, and Ki67 in determining the clinical significant lesions and the results are summarized in Table 3. Geminin showed higher specificity (86.7%) than p16 (75.6%) and Ki67 (64.4%). The sensitivity of geminin, p16, and Ki67 was 90%, 87.5%, and 95%, respectively. The PPV was higher for geminin (85.7%) than for p16 (76.1%) and Ki67 (70.3%). The NPV was comparable for all these 3 markers. In addition, the accuracy of geminin was higher (88.2%) than p16 (81.2%) and Ki67 (78.8%). Among the 85 Chinese patients with CIN, 76 (89.4%) were found HPV-positive and 9 (10.6%) HPV-negative. The relationship between HPV status and geminin or p16 expression was analyzed as summarized in Table 4. Geminin expression pattern exhibited a weak correlation with HPV status (correlation coefficient = 0.264, *P* = .015). However, no correlation was found between p16 expression and HPV status (correlation coefficient = 0.144, *P* = .190).

**Table 3:**
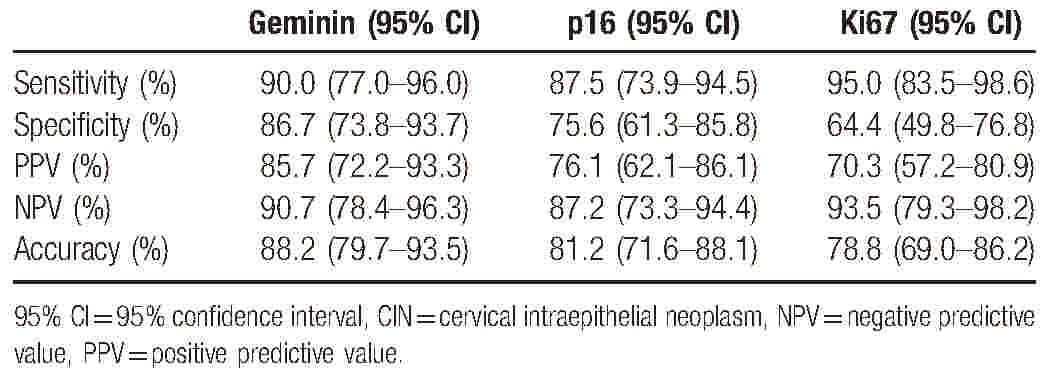
Sensitivity, specificity, PPV, NPV, and accuracy for differentiation between CIN1 and CIN2/3.

**Table 4:**
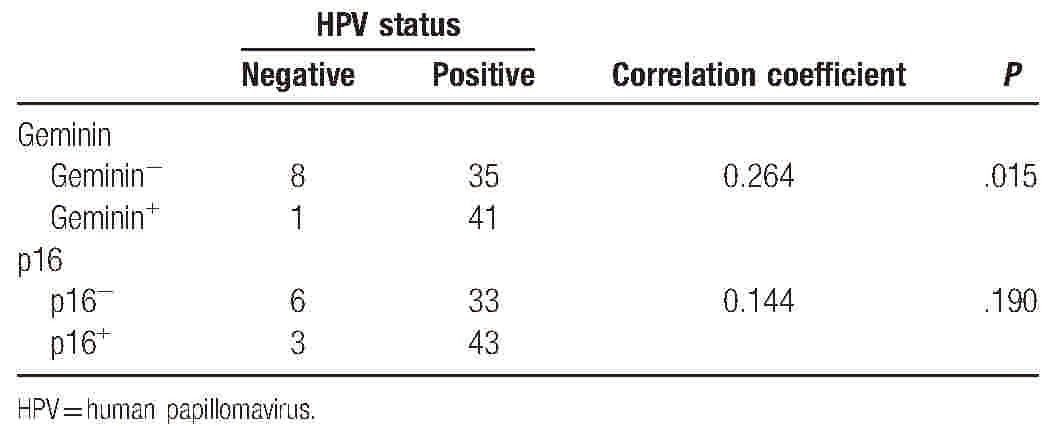
Correlation between geminin or p16 expression and HPV status.

## 4 Discussion

The aim of this study was to assess whether geminin could be a biomarker to discriminate cervical high-grade lesions. Our results showed that geminin had a high degree of sensitivity and specificity in determining CIN2/3. The specificity of geminin (86.7%) exceeded that of the commonly used biomarkers p16 (75.6%) and Ki67 (64.4%). The sensitivity of geminin (90.0%) was higher than that of p16 (87.5%), but lower than that of Ki67 (95.0%).

Accurate histological grading of CIN was clinically important, because CIN2 and CIN3 were regarded as precursors of invasive cervical carcinomas and therapy was indicated. Histopathology is a gold standard for diagnosis of CIN. However, the interobserver and intraobserver variabilities in interpreting cervical biopsy specimen are relatively high.^[1]^ Therefore, it is still challenging to discriminate between CIN1 and CIN2/3.

The commonly used immunohistochemical markers p16 and Ki67 were not very accurate to help for the distinction between CIN1 and CIN2/3.^[18]^ Furthermore, these 2 markers were highly sensitive to detect the presence of CIN2/3, but their specificity was relatively low in our study. This is in accordance with many previous studies.^[19,20]^ Our results demonstrated that the specificity, PPV, and accuracy of geminin were highest among these biomarkers. In addition, geminin expression is significantly increased in CIN2/3 compared with CIN1. This implied that the use of geminin immunohistochemical analysis might be a surrogate marker of high-grade cervical intraepithelial neoplasia.

High-risk HPV persistent infection is significantly associated with CIN2/3 and invasive cervical carcinomas.^[21]^ In the present study, HPV positivity was detected in 82.2% of CIN1 and 97.5% of CIN2/3. Our results were higher than those in a recent study.^[22]^ A 4-year surveillance study by Zhang et al^[22]^ have reported that the prevalence of HPV increased with cervical lesions severity and the HPV positivity rates were 72.4% for CIN1, 81.4% for CIN2, and 88.1% for CIN3. The discrepancy might be caused by our small sample size. Moreover, the average age of patients in our study was 47.8 ± 7.9 years, which might be another reason for the higher HPV positivity rates because age was an important factor for high-risk HPV infection clearance.^[23]^ In the present study, p16 expression was not associated with HPV status. The finding is in accordance with previous studies.^[24,25]^ No correlation was found between p16 and HPV status in esophageal squamous cell carcinoma in Kazakh population by Wang et al,^[25]^ thus suggesting that p16 might be an unreliable surrogate marker for HPV status. Nevertheless, p16 expression was previously reported to exhibit a correlation with HPV status.^[26]^ The inconsistency might be attributed to absence of uniformity in cutoff value and variation in HPV status among patients from different geographic origins.^[27]^ Interestingly, a weak correlation between geminin and HPV status was observed in our study.

There are some limitations in our study. First, the sample size of our study is relatively small. In addition, the cutoff value for increased geminin expression used in our study needs to be validated in an independent set of cervical samples. Another limitation in this study is that we focused on the expression of geminin in CIN, but did not evaluate geminin expression in cervical carcinomas. Our results suggested that geminin might serve as a diagnostic biomarker for identifying CIN2/3; however, we are not informed about its clinical use as a prognostic variable in cervical carcinomas. Furthermore, additional mechanistic studies to investigate the role of geminin in the pathogenesis of CIN progress are needed.

In conclusion, geminin is a highly sensitive and specific biomarker for CIN diagnosis. It can be used in addition to p16 and Ki67 staining in routine pathology practice when discriminating between CIN1 and CIN2/3 is difficult.

## 5 Conclusion

**Figure 1:**
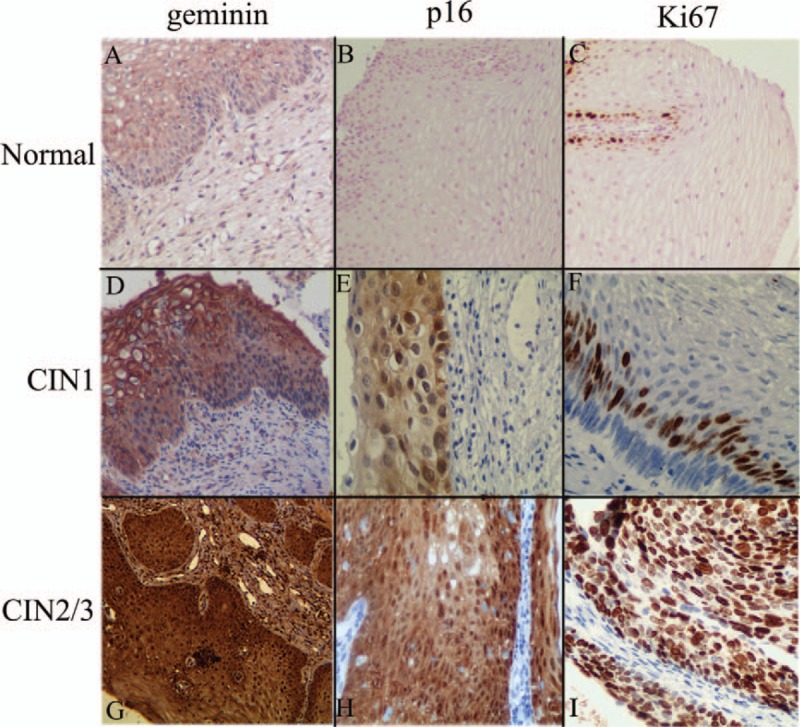
Representative immunohistochemical images of geminin (A, D, and G, ×100), p16 (B, ×100, E and H, ×200), and Ki67 (C, ×100, F and I, ×200) expression in normal tissues, CIN1, and CIN2/3. All pictures were taken at original magnification.

Our study showed that the immunoquantification of geminin appeared to be a new marker for the differentiation between CIN1 and CIN2/3 when there is doubt about the grading of CIN. Nonetheless, further studies are necessary before implementing geminin in clinical practice (Fig. 1).

## Acknowledgment

We would like to thank all the subjects for their participation in this research, as well as the clinical coworkers form the department of Obstetrics and Gynecology.

## References

[1] Martin CM O’Leary JJ Phil D Histology of cervical intraepithelial neoplasia and the role of biomarkers. Best Pract Res Clin Obstet gynaecol 2011;25:605–15. 21636328 10.1016/j.bpobgyn.2011.04.005

[2] Castle PE Schiffman M Wheeler CM Evidence for frequent regression of cervical intraepithelial neoplasia-grade 2. Obstet Gynecol 2009;113:18–25. 19104355 10.1097/AOG.0b013e31818f5008 PMC2694845

[3] McCredie MR Sharples KJ Paul C Natural history of cervical neoplasia and risk of invasive cancer in women with cervical intraepithelial neoplasia 3: a retrospective cohort study. Lancet Oncol 2008;9:425–34. 18407790 10.1016/S1470-2045(08)70103-7

[4] Lobato S Tafuri A Fernandes PÁ Minichromosome maintenance 7 protein is a reliable biological marker for human cervical progressive disease. J gynecol Oncol 2012;23:11–5. 22355461 10.3802/jgo.2012.23.1.11 PMC3280060

[5] Safaeian M Schiffman M Gage J Detection of precancerous cervical lesions is differential by human papillomavirus type. Cancer Res 2009;69:3262–6. 19351830 10.1158/0008-5472.CAN-08-4192 PMC3155840

[6] Wright TC Stoler MH Behrens CM Primary cervical cancer screening with human papillomavirus: end of study results from the ATHENA study using HPV as the first-line screening test. Gynecol Oncol 2015;136:189–97. 25579108 10.1016/j.ygyno.2014.11.076

[7] Nogueira MC Guedes Neto Ede P Rosa MW Immunohistochemical expression of p16 and p53 in vulvar intraepithelial neoplasia and squamous cell carcinoma of the vulva. Pathol Oncol Res 2006;12:153–7. 16998595 10.1007/BF02893362

[8] Léonard B Kridelka F Delbecque K A clinical and pathological overview of vulvar condyloma acuminatum, intraepithelial neoplasia, and squamous cell carcinoma. Biomed Res Int 2014;2014:480573. 24719870 10.1155/2014/480573 PMC3956289

[9] Sari Aslani F Safaei A Pourjabali M Evaluation of Ki67, p16 and CK17 markers in differentiating cervical intraepithelial neoplasia and benign lesions. Iran J Med Sci 2013;38:15–21. 23645953 PMC3642940

[10] McCluggage WG Premalignant lesions of the lower female genital tract: cervix, vagina and vulva. Pathology 2013;45:214–28. 23442737 10.1097/PAT.0b013e32835f21b1

[11] Kushwaha PP Rapalli KC Kumar S Geminin a multi task protein involved in cancer pathophysiology and developmental process: a review. Biochimie 2016;131:115–27. 27702582 10.1016/j.biochi.2016.09.022

[12] Guo J Sun N Cell cycle regulator geminin is dispensable for the proliferation of vascular smooth muscle cells. Sci China Life Sci 2013;56:731–8. 23838810 10.1007/s11427-013-4513-1

[13] Yagi T Inoue N Yanai N Prognostic significance of geminin expression levels in Ki67-high subset of estrogen receptor-positive and HER2-negative breast cancers. Breast Cancer 2016;23:224–30. 25082658 10.1007/s12282-014-0556-9

[14] Shomori K Nishihara K Tamura T Geminin, Ki67, and minichromosome maintenance 2 in gastric hyperplastic polyps, adenomas, and intestinal-type carcinomas: pathobiological significance. Gastric Cancer 2010;13:177–85. 20820987 10.1007/s10120-010-0558-z

[15] Haruki T Shomori K Hamamoto Y Geminin expression in small lung adenocarcinomas: implication of prognostic significance. Lung Cancer 2011;71:356–62. 20650542 10.1016/j.lungcan.2010.06.013

[16] Zhu W DePamphilis ML Selective killing of cancer cells by suppression of geminin activity. Cancer Res 2009;69:4870–7. 19487297 10.1158/0008-5472.CAN-08-4559 PMC2749580

[17] Martin CM Astbury K McEvoy L Gene expression profiling in cervical cancer: identification of novel markers for disease diagnosis and therapy. Methods Mol Biol 2009;511:333–59. 19347305 10.1007/978-1-59745-447-6_15

[18] Lorenzato M Caudroy S Bronner C Cell cycle and/or proliferation markers: what is the best method to discriminate cervical high-grade lesions? Hum Pathol 2005;36:1101–7. 16226110 10.1016/j.humpath.2005.07.016

[19] Hariri J Oster A The negative predictive value of p16INK4a to assess the outcome of cervical intraepithelial neoplasia 1 in the uterine cervix. Int J Gynecol Pathol 2007;26:223–8. 17581402 10.1097/01.pgp.0000236942.51840.56

[20] Cones-Zamora P Domenech-Peris A Orantes-Casado FJ Effect of human papillomavirus on cell cycle-related protein P16, Ki-67, Cyclin D1, P53 and ProEx C in precursor lesion of cervical cancinoma. Am J Clin Pathol 2009;132:378–90. 19687314 10.1309/AJCPO0WY1VIFCYDC

[21] Abate E Aseffa A El-Tayeb M Genotyping of human papillomavirus in paraffin embedded cervical tissue samples from women in Ethiopia and the Sudan. J Med Virol 2013;85:282–7. 23160919 10.1002/jmv.23437

[22] Zhang L Bi Q Deng H Human papillomavirus infections among women with cervical lesions andcervical cancer in Eastern China: genotype-specific prevalence and attribution. BMC Infect Dis 2017;17:107. 28143439 10.1186/s12879-017-2223-1 PMC5282745

[23] Zhang G Lang J Shen K High-risk human papillomavirus infection clearance following conization among patients with cervicalintraepithelial neoplasm grade 3 aged at least 45 years. Int J Gynaecol Obstet 2017;136:47–52. 10.1002/ijgo.12000 28099704

[24] Galmiche L Coste-Burel M Lopes P The expression of p16 is not correlated with HPV status in CINI. Histopathology 2006;48:767. 10.1111/j.1365-2559.2006.02340.x 16681697

[25] Wang L Li J Hou J P53 expression but not p16(INK4A) correlates with human papillomavirus-associated esophageal squamous cell carcinoma in Kazakh population. Infect Agents Cancer 2016;11:19. 27076841 10.1186/s13027-016-0065-x PMC4830030

[26] Liu SZ Zandberg DP Schumaker LM Correlation of p16 expression and HPV type with survival in oropharyngeal squamous cell cancer. Oral Oncol 2015;51:862–9. 26183400 10.1016/j.oraloncology.2015.06.014

[27] Syrjaenen K Geographic origin is a significant determinant of human papillomavirus prevalence in oesophageal squamous cell carcinoma: systematic review and meta-analysis. Scand J Infect Dis 2013;45:1–8. 22830571 10.3109/00365548.2012.702281

